# The Effect of Retirement on Cardiovascular Disease: Evidence From European Countries

**DOI:** 10.1093/geroni/igaf122.1342

**Published:** 2025-12-31

**Authors:** Jiaru Liu

**Affiliations:** King’s College London, London, England, United Kingdom

## Abstract

Retirement marks a pivotal life stage for many individuals. As older adults transition into retirement, changes in lifestyle, socioeconomic status, income, and social relationships can significantly influence their health and well-being. This study investigates the short- and lagged-term relationship between retirement and cardiovascular disease (CVD) - one of the most commonly diagnosed conditions among older adults. Using individual-level data from the Survey of Health, Ageing, and Retirement in Europe (SHARE), we employ an Instrumental Variable Fixed Effects (IV-FE) approach to estimate both the immediate and lagged impact of retirement on CVD and further explore the underlying mechanisms and potential heterogeneity in effects. Our findings suggest that retirement does not exert a significant impact on heart attacks in the short term, but the probability of having a heart attack increases by almost 7% within two years. We further explore several underlying mechanisms through which retirement may influence CVD. While we found no significant associations between metabolic or behavioral risk factors and CVD as potential mediators, our findings highlight a notable exception: a decline in mental health, a key psychosocial risk factor, appears to significantly contribute to adverse cardiovascular outcomes following retirement. This highlights the importance of incorporating mental health support and social engagement initiatives into retirement planning and community programs for older adults.

